# Congenital Pouch Colon associated with Pseudoexstrophy: Report of Two Cases

**Published:** 2016-01-01

**Authors:** Praveen Jhanwar, Nand Kishor Shinde, Jigar N Patel, Nitin Pant, Rajiv Chadha

**Affiliations:** Department of Pediatric Surgery, Lady Harding Medical College and Kalawati Saran Children’s Hospital, New Delhi-110001

**Keywords:** Anorectal malformation, Pouch colon, Pseudoexstrophy, Meckel's diverticulum, Hemiuterus

## Abstract

This report describes two newborn girls with single perineal opening (cloaca), and pseudoexstrophy in the form of divergent pubic bones and rectus muscles, and a low-set umbilicus. Both patients had a type II congenital pouch colon (CPC) with one hemiuterus and vagina on each side in the pelvis. In one patient, a Meckel’s diverticulum was present 5 cm from the ileocecal junction. In both girls, a diverting proximal ileostomy was the initial surgery.

## CASE REPORT

**Case 1**

A 20-day old full-term girl, weighing 3.1 kg, was brought with complaints of passing urine and stools from a single perineal opening. The baby was feeding normally and thriving. There was no significant antenatal history. Examination revealed the stigmata of pseudoexstrophy with a low-set umbilicus, scarring around the umbilicus, and wide pubic diastasis (Fig. 1). Perineal examination showed a wide cloacal opening along with folds radiating inwards and upwards from the margins of the opening. The perineum was well-developed. The rest of the clinical examination was normal. Hematological and biochemical parameters, including renal function tests were within normal limits. Plain X-ray abdomen showed a large air-fluid level on the left side of the abdomen and a normal sacrum. Abdominal ultrasound (US) showed normal kidneys. Laparotomy revealed a type II CPC with 5 cm of normal colon, a single appendix, and one hemiuterus and vagina flanking the lower terminal portion of the colonic pouch on each side. A divided ileostomy was constructed proximal to the colonic pouch. Postoperative recovery was uneventful.

**Figure:**
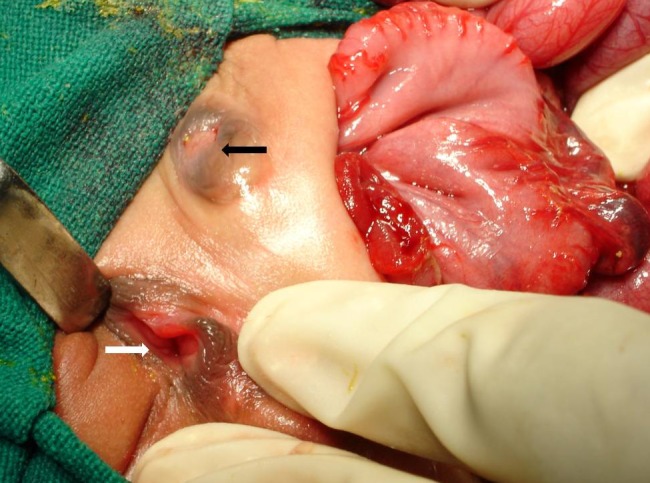
Figure 1:Photograph at surgery showing the type II colonic pouch, the external genitalia (white arrow) and the low-set umbilicus with scarring around it (black arrow).

**Case 2**

A preterm (32 weeks gestation with 1.814 kg weight) girl was brought soon after birth with a single perineal opening. Clinical and radiological examination (Fig. 2) showed similar findings as reported in the first case except for mild hydronephrosis of the left kidney and a distended urinary bladder on ultrasound abdomen. Laparotomy revealed type II CPC with a distended thin-walled colonic pouch, a single appendix, a distended flaccid bladder, and one hemiuterus and vagina on each side in the pelvis, flanking the lower terminal portion of the colonic pouch (Fig. 3). A Meckel’s diverticulum was present just 4-5 cm proximal to the cecum. The grossly distended pouch was decompressed by a small incision on its anterior surface which was closed subsequently. A divided ileostomy was constructed proximal to the pouch and a suprapubic cystostomy performed with a No. 10 Fr Foley’s catheter. Postoperative recovery was uneventful and the child is at present awaiting further corrective surgery.

**Figure:**
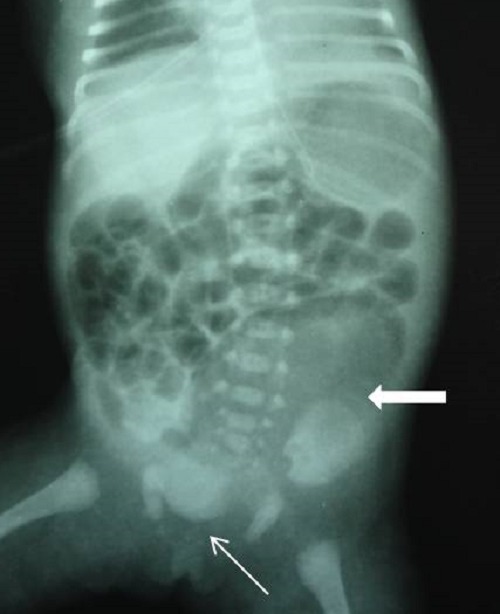
Figure 2:Infantogram showing the large gas shadow of the colonic pouch in the left side of the abdomen (arrow). Note the wide pubic diastasis (fine arrow).

**Figure:**
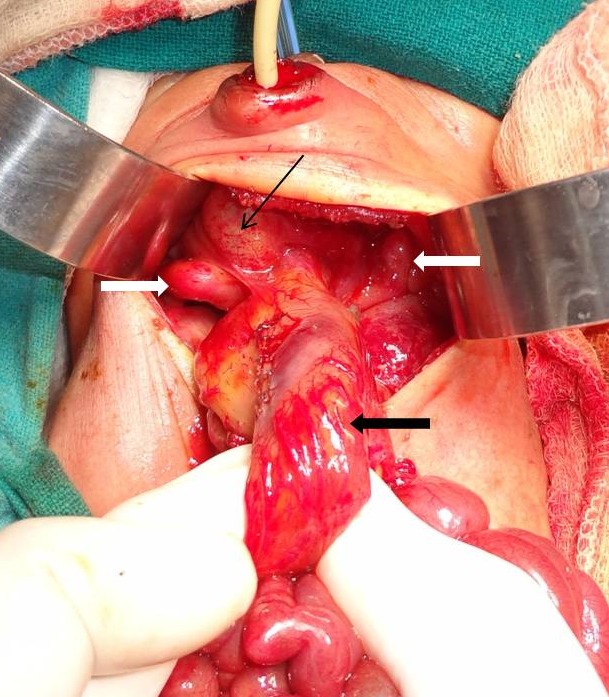
Figure 3:Photograph at surgery showing the colonic pouch that had been decompressed (black arrow). Note the hemiuterus on each side in the pelvis (white arrows) and the lobulated, flaccid urinary bladder (fine arrow).

## DISCUSSION

Pseudoexstrophy or covered exstrophy is a variant of classical vesical exstrophy in which all the usual musculoskeletal findings of classical exstrophy are present but the bladder is closed with varying degrees of skin and subcutaneous cover [1-3]. Abnormalities of the external genitalia are frequent and these include a bifid clitoris in 50 % girls, a small laterally displaced penis, partial diphallus with incomplete urethral duplication, and two widely separated scrotal halves [4-6]. Unlike classical exstrophy, abnormalities of the upper urinary tract like vesicoureteric reflux or renal dysplasia have been reported [4, 7], and an associated ARM may be present [4, 7].

There are 2 reports in the literature of patients of CPC with associated pseudoexstrophy [1, 2]. Chadha et al [1] described a male newborn with similar pseudoexstrophy and type IV CPC. Herman et al [2] reported a newborn girl with type II CPC and pseudoexstrophy associated with various spine, spinal cord, GIT, and genitourinary anomalies.

Exstrophy variants are explained by incomplete rupture or persistence of an abnormal infraumbilical cloacal membrane which acts as a wedge and prevents the lateral mesoderm from organizing and progressing medially between the ectodermal and endodermal layers [4]. CPC is believed to result from a very early arrest in partitioning of the cloaca by the urorectal septum (URS) [8] which may be due to early errors in organization, growth, and proliferation of the mesenchyme in the region of the URS. If this mesodermal defect also affects the mesenchyme in the adjacent developing hindgut, ‘defective organogenesis’ of the hindgut at an early stage before it lengthens could account for later development of the colonic pouch with its unique anatomic and physiological characteristics [8,9]. Significantly, especially in reports from India, CPC is infrequently associated with major malformations in other organ systems and the sacrum is usually normal [8,9]. There is a high incidence of a Meckel’s diverticulum [8,9] with sometimes, as in one of our cases, the diverticulum being close to the ileocecal junction [8]. This suggests that the abnormal development and shortening may affect the entire post-arterial limb of the midgut and hindgut [8]. It is tempting to suggest, therefore, that if the defective differentiation and growth of the mesenchyme in the URS and adjacent hindgut is associated with a similar mesodermal defect in the infraumbilical region of the ventral body wall, a combination of CPC and pseudoexstrophy may result. The multiple anomalies in the case reported by Herman et al [2] and in some cases of pseudoexstrophy reported in the literature [4, 6, 7] may be because in these cases, the abnormality was part of a more generalized error in mesodermal development and differentiation in the caudal developmental field.

## Footnotes

**Source of Support:** Nil

**Conflict of Interest:** None declared

